# Sex differences in campylobacteriosis incidence rates at different ages - a seven country, multi-year, meta-analysis. A potential mechanism for the infection

**DOI:** 10.1186/s12879-020-05351-6

**Published:** 2020-08-25

**Authors:** Manfred S. Green, Naama Schwartz, Victoria Peer

**Affiliations:** School of Public Health, University of Haifa, Abba Khoushy 199, Mount Carmel, 3498838 Haifa, Israel

**Keywords:** Campylobacteriosis, Sex differences, Incidence rate ratios, Meta-analysis, Male predominance

## Abstract

**Background:**

There is evidence that males have higher incidence rates (IR) of campylobacteriois than females. The objectives of this study were to determine whether these observations differ between age groups and are consistent over different countries and during different time periods.

**Methods:**

We obtained data on IRs of campylobacteriosis by sex and age group over a period of 11–26 years from seven countries. Male to female incidence rate ratios (IRR) were computed by age group, country and time period. For each age group, we used meta-analytic methods to combine the IRRs. Sensitivity analysis was used to test whether the results are robust to differences between countries and time periods. Meta-regression was conducted to estimate the different effects of age, country, and time period on the IRR.

**Results:**

In the age groups < 1, 1–4, 5–9, 10–14, 15–44, 45–64 and 65+ years old, the pooled IRRs (with 95% CI) were 1.31 (1.26–1.37), 1.34 (1.31–1.37), 1.35 (1.32–1.38), 1.73 (1.68–1.79), 1.10 (1.08–1.12), 1.19(1.17–1.21) and 1.27 (1.24–1.30), respectively. For each age group, the excess campylobacteriosis IRs in males differed at different age groups. However, despite some quantitative differences between countries, the excess was consistently present over long time-periods. In meta-regression analysis, age group was responsible for almost all the variation in the IRRs.

**Conclusions:**

The male predominance in campylobacteriosis IRs starts in infancy. This suggests that this is due, at least in part, to physiological or genetic differences and not just behavioural factors. These findings can provide clues to the mechanisms of the infection and could lead to more targeted treatments and vaccine development.

## Key messages

- This study provides stable estimates of the extent and consistency of the excess male campylobacteriosis incidence rates in different age groups.
- The mechanism underlying the excess in males is still largely unknown. The consistency of the findings in infants and young children tends to exclude factors related only to differences in exposure.
- Our findings should stimulate investigations on genetic and hormonal determinants of campylobacteriosis in particular and infectious diseases in general.
- This study reinforces the need to consider sex differences when developing and testing new treatments and vaccines.

## Background

Campylobacteriosis, usually caused by the non-spore-forming, gram-negative bacterium, Campylobacter jejuni (C. jejuni) is one of the most common causes of bacterial gastroenteritis [1]. The disease can be debilitating, with occasional severe complications such as toxic mega-colon, sepsis and Guillane-Barré syndrome [1]. Based on serosurveys, the subclinical incidence of campylobacteriosis is much higher than the incidence of clinically overt disease [2]. Transmission is mainly food-borne, particularly from uncooked poultry products [3]. Fecal-oral transmission among humans can occur such as through person-to-person sexual contact [4] and contamination by food handlers and it is a common cause of travellers diarrhea [3, 5]. The mechanism of campylobacteriosis infection is complex and the variability of disease outcomes is thought to be linked to the immune response induced by the bacteria [6]. The virulence factors induce a pro-inflammatory response that is initiated by the intestinal epithelial cells, propagated by innate immune cells and modulated by the cells of the adaptive immune response [6]. Mortality rates from gram-negative sepsis appears to be lower in females [7] and in animal studies, Zeng et al. [8] has demonstrated a more efficient immune response to gram-negative bacteria in female mice, although this may not be directly extrapolated to humans.

Sex differences in the incidence rates for infectious diseases has frequently been described [9–13]. There are reports indicating higher campylobacteriosis incidence rates in males, based on population data from multiple [10] and single countries [11, 12]. However, pooled estimates of age-specific sex differences in the incidence rates of the disease over time have not been rigorously evaluated in large, representative databases with reliable denominators. This information could improve our understanding of the mechanism of the host response to the infection. In the present study, we analysed the magnitude and consistency of the sex differences in the incidence of campylobacteriosis in different age groups, for a number of countries and time periods, using national data.

## Methods

The methods used to collect the data are similar to those that we have described in previous publications on sex differences in infectious diseases [13, 14]. Data on reported cases of campylobacteriosis by age, sex and calendar year were obtained from relevant government institutions for seven countries. In order to ensure uniform data quality, we only included countries for which campylobacteriosis is a notifiable disease, have reliable reporting systems and use modern laboratory methods for diagnosis of the disease. In addition, we required that the countries included provide access to data by age and sex over a period of several years. We received administrative permission from the official representative of Israeli Ministry of Health to use the data for publication. Data for Australia were obtained from the National Notifiable Diseases Surveillance System (NNDSS) [15], for Canada from Public Health Agency of Canada (PHAC) [16], for Finland from the National Institute for Health and Welfare (THL) [17], for Germany from the German Federal Health Monitoring System [18], for Israel from the Ministry of Health, for New Zealand, from the Institute of Environmental Science and Research (ESR) [19], and for Spain from the Spanish Epidemiological Surveillance Network [20]. Information about the population size by age, sex and year was obtained for Australia from ABS. Stat (Australian Bureau of Statistics) [21], for Canada from Statistics, Canada database [22], for Finland from the Statistics Finland’s PX-Web databases [23], for Germany from the German Federal Health Monitoring System [24], for Israel from Central Bureau of Statistics [25], for New Zealand from Statistics New Zealand [26], and for Spain from the Demographic Statistics Database [27].

### Statistical analyses

Campylobacter IRs per 100,000 were calculated by sex, age group, for each country and calendar year using the number of reported cases divided by the respective population size and multiplied by 100,000. The age groups considered were < 1 (infants), 1–4 (early childhood), 5–9 (late childhood), 10–14 (puberty), 15–44 (young adulthood), 45–64 (middle adulthood) and 65+ (senior adulthood) years old. The surveillance systems in Canada and New Zealand used similar age-groups except for the following: 15–39, 40–59 and 60 + .

Australia and Finland do not report separately for ages 0–1 and 1–4 and were excluded from the analyses in these age groups. The male to female incidence rate ratios (IRR), for each age group, country and time period, were calculated by dividing the incidence rate in males by those for females.

As in previous studies [13, 14], we used meta-analytic methodology to evaluate the overall magnitude of the sex differences in the incidence of campylobacteriosis by age group, across different countries and over a number of years. The outcome variable was the male to female IRR. The data presented (forest plots) are the IRRs by age group, for 2 years intervals for each country. The reported period for all countries was between 1991 and 2016, divided into 2 year intervals (aside from data for each individual year). For each age group, the IRRs for each country were pooled over time periods and then the pooled IRRs for each country were combined. As for standard meta-analysis, heterogeneity between groups was evaluated using Cochran’s Q statistic, Tau^2^ and I^2^. The statistics were used to assist in deciding on the use of the fixed or random effects model for pooling the IRRs. If the Q test yielded a *p* < 0.1, and/or I^2^ ≥ 50%, the random effects model [28] was always used to estimate pooled IRRs and the associated 95% confidence intervals (CI). In other cases, if there still appeared to be considerable heterogeneity, the random effects model was preferred. We performed leave-one-out sensitivity analysis and recomputed the pooled IRRs, in order to evaluate the effect of individual county and reported years on the risk of campylobacteriosis,. We performed the Egger test for asymmetry for testing for a possible imbalance in the studies around the pooled IRR.

In order to test for the contributions of age-group, country and time period, to the variability in the IRRs, we carried out meta-regression analyses. In these analyses, the IRR was the dependent variable, and age-group, country and time-period were entered as possible explanatory variables. The meta-analyses and meta-regressions were carried out using STATA software version 12.1 (Stata Corp., College Station, TX).

## Results

### Descriptive statistics

The summary of male and female incidence rates (per 100,000 populations) in different countries for each age group and relevant years is presented in Table A1, Appendix A. In every country, and in all age groups the incidence rates of Campylobacter were higher in males compared to females.

### Meta-analyses by age group and group of calendar years

**Fig. 1:**
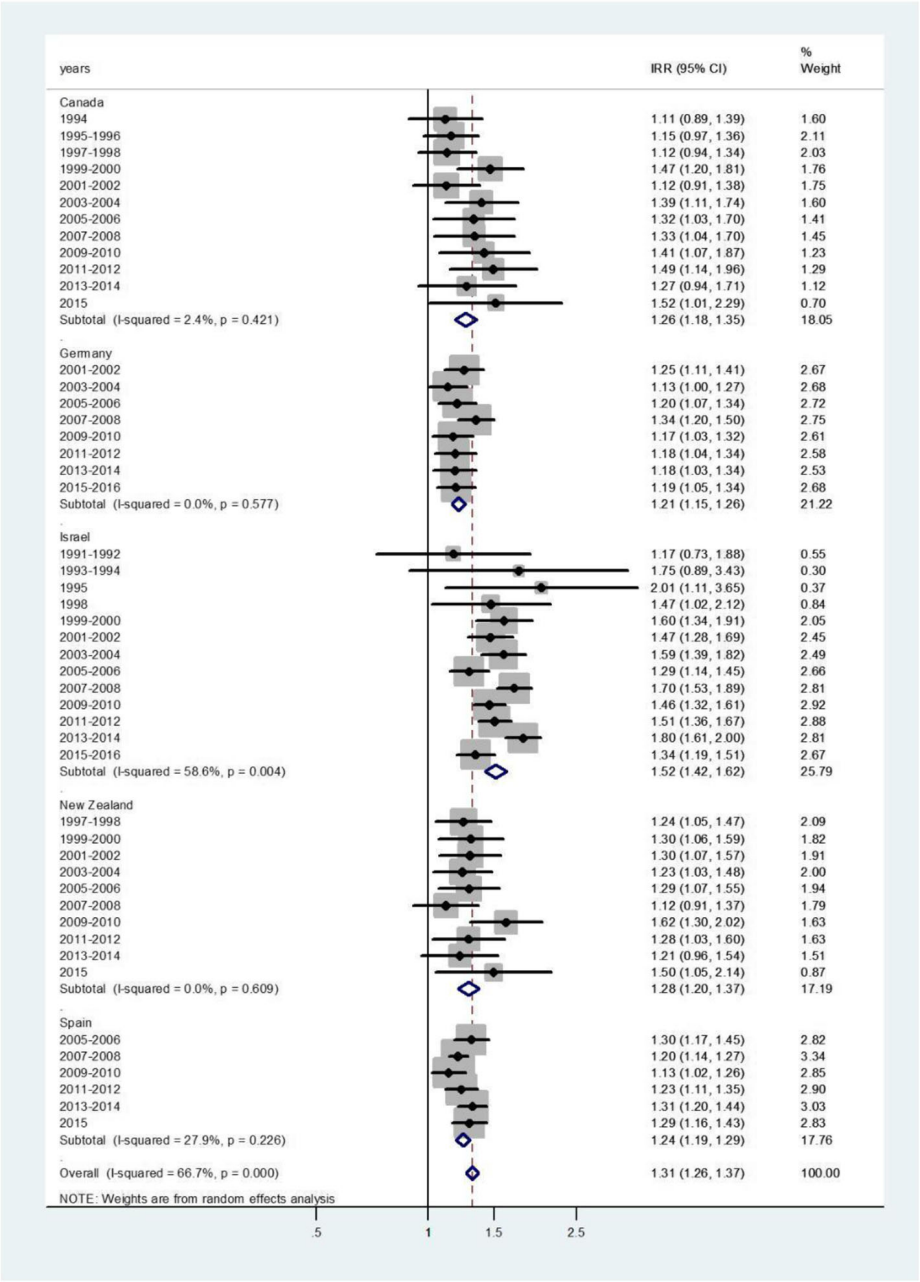
Forest plot of the male to female campylobacteriosis IRR for infants (< 1 year), for different years, in Canada, Germany, Israel, New Zealand, and Spain

**Fig. 2:**
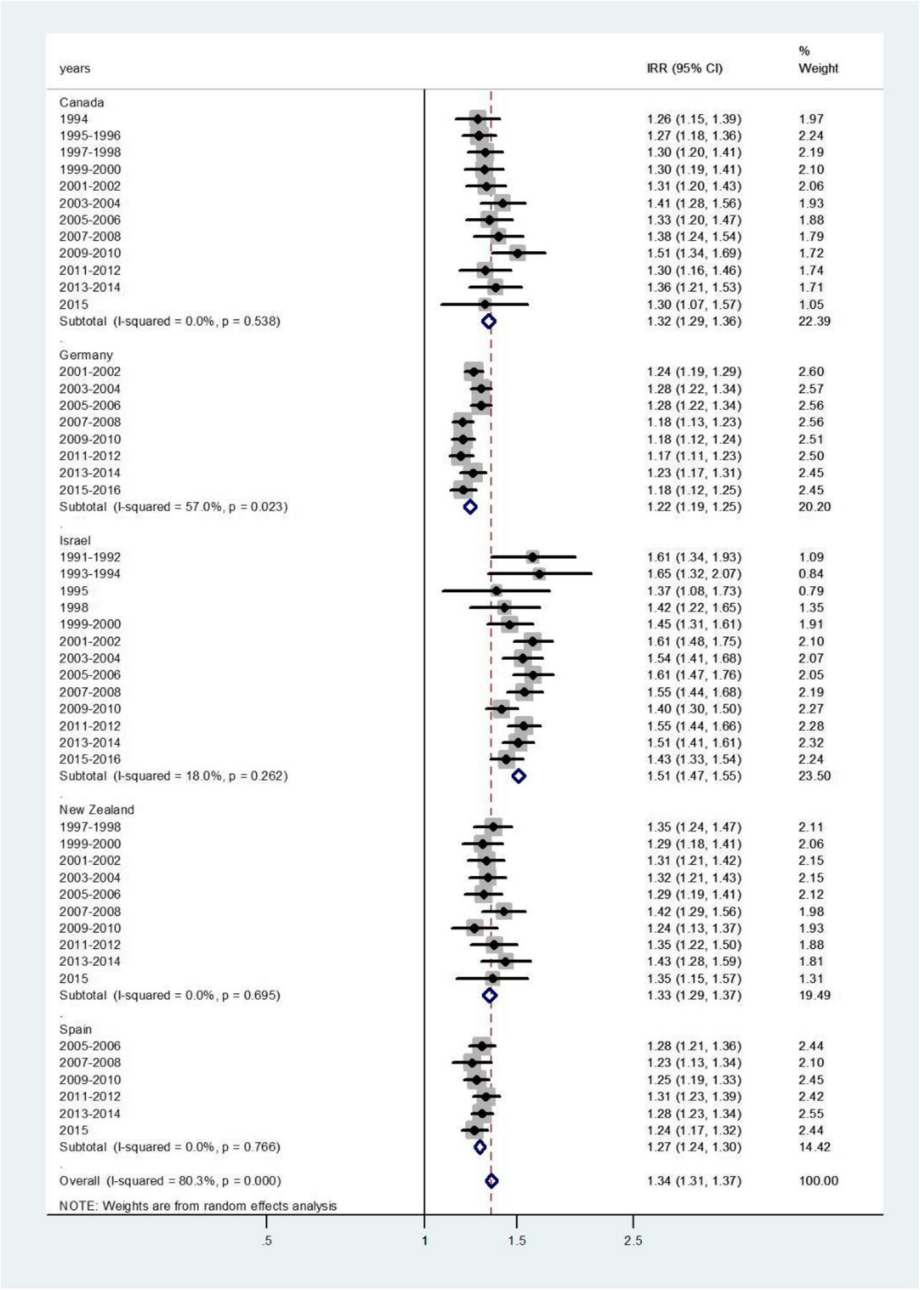
Forest plot of the male to female campylobacteriosis IRR at ages 1–4, for different years, in Canada, Germany, Israel, New Zealand, and Spain

**Fig. 3:**
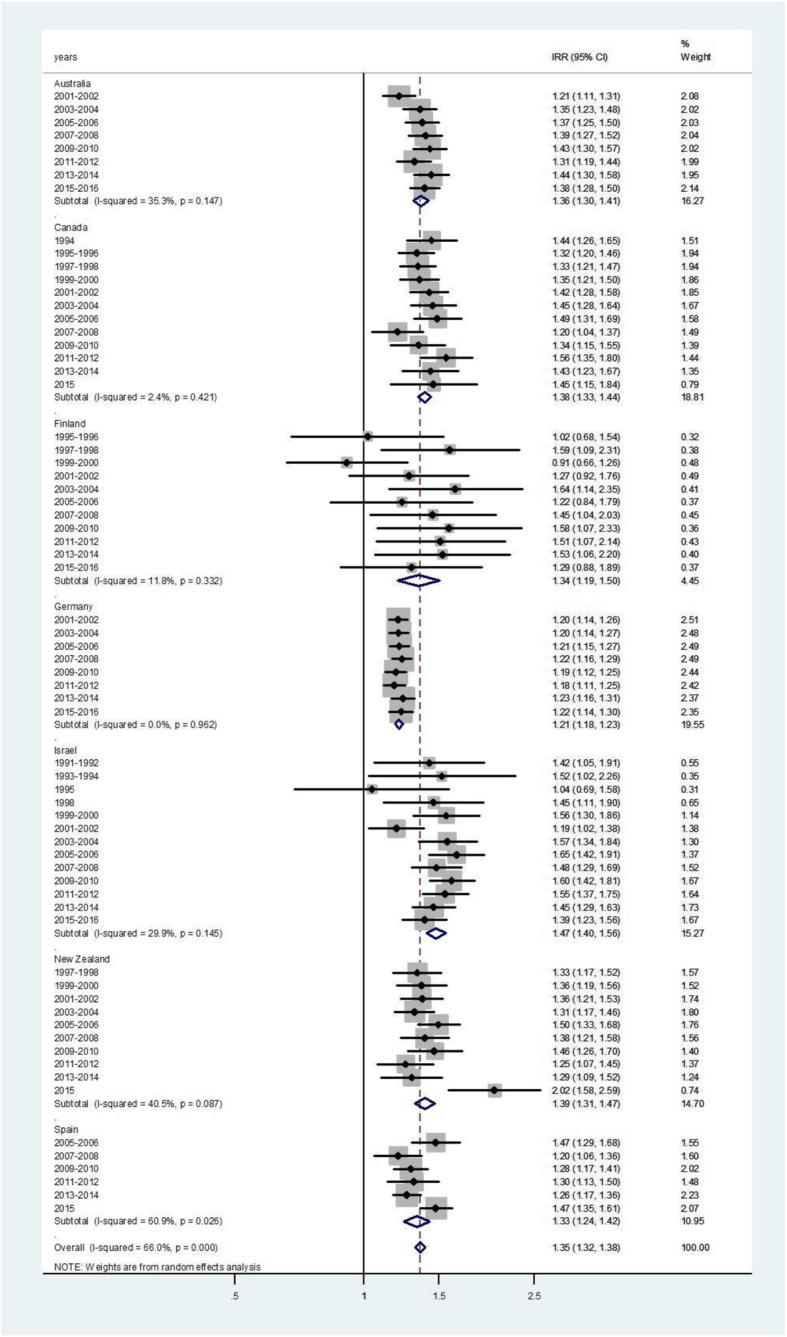
Forest plot of the male to female campylobacteriosis IRR at ages 5–9, for different years, in Australia, Canada, Finland, Germany, Israel, New Zealand, and Spain

**Fig. 4:**
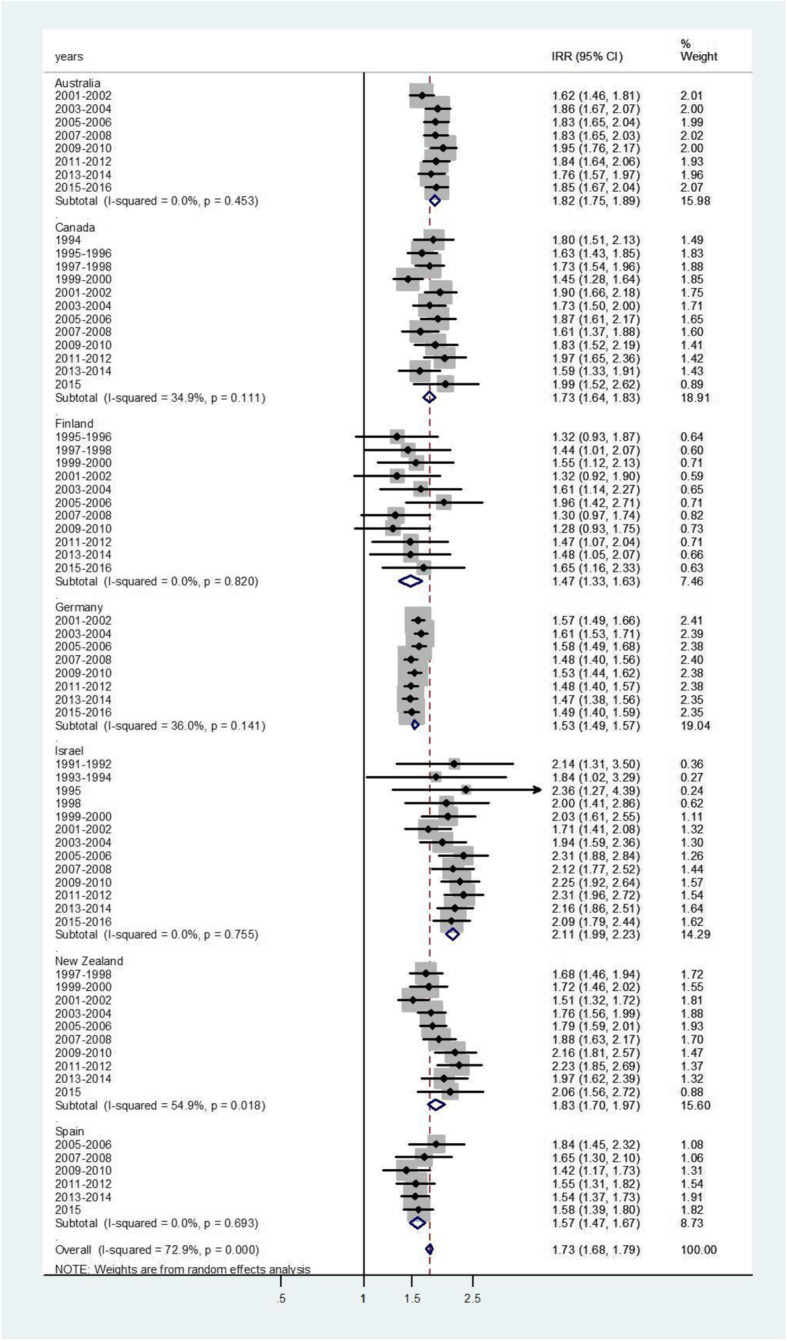
Forest plot of the male to female campylobacteriosis IRR at ages 10–14 for different years, in Australia, Canada, Finland, Germany, Israel, New Zealand, and Spain

**Fig. 5:**
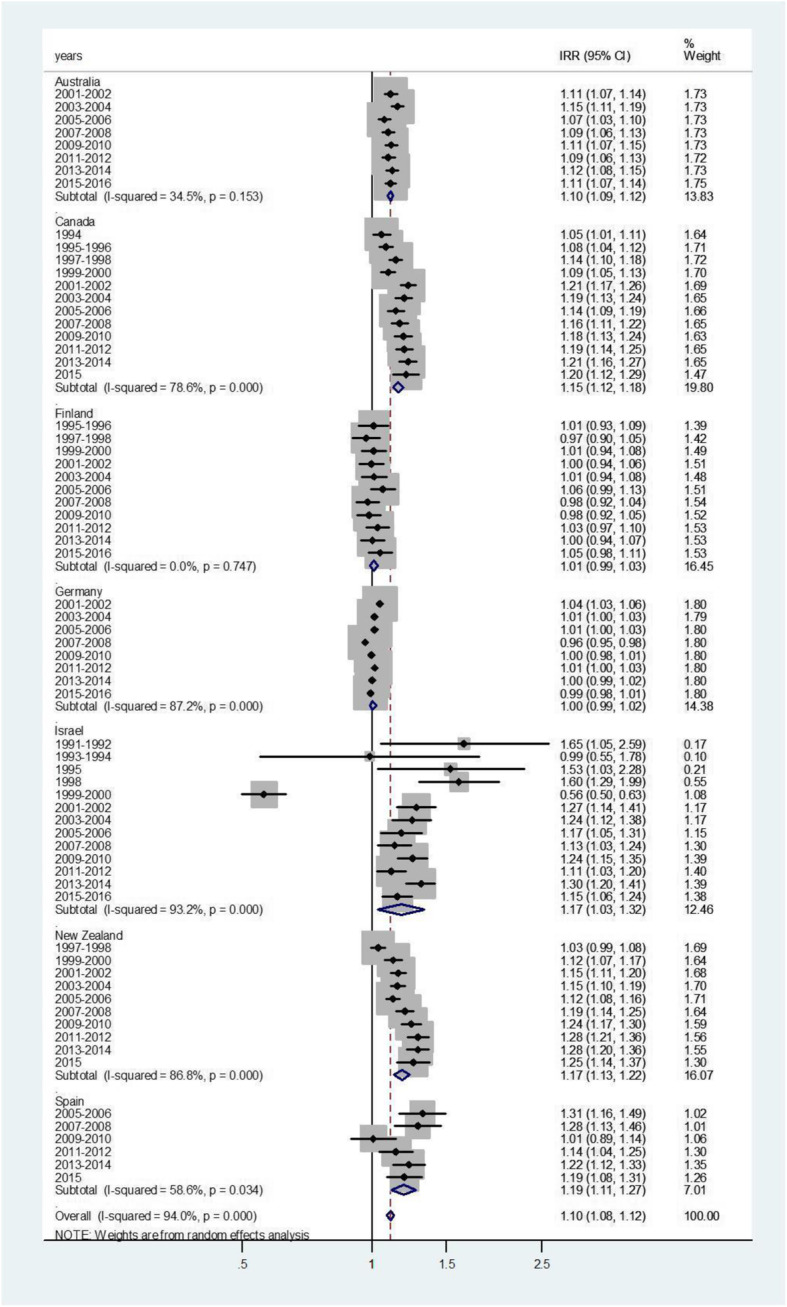
Forest plot of the male to female campylobacteriosis IRR at ages 15–44 or 15–39, for different years, in Australia, Canada, Finland, Germany, Israel, New Zealand, and Spain

**Fig. 6:**
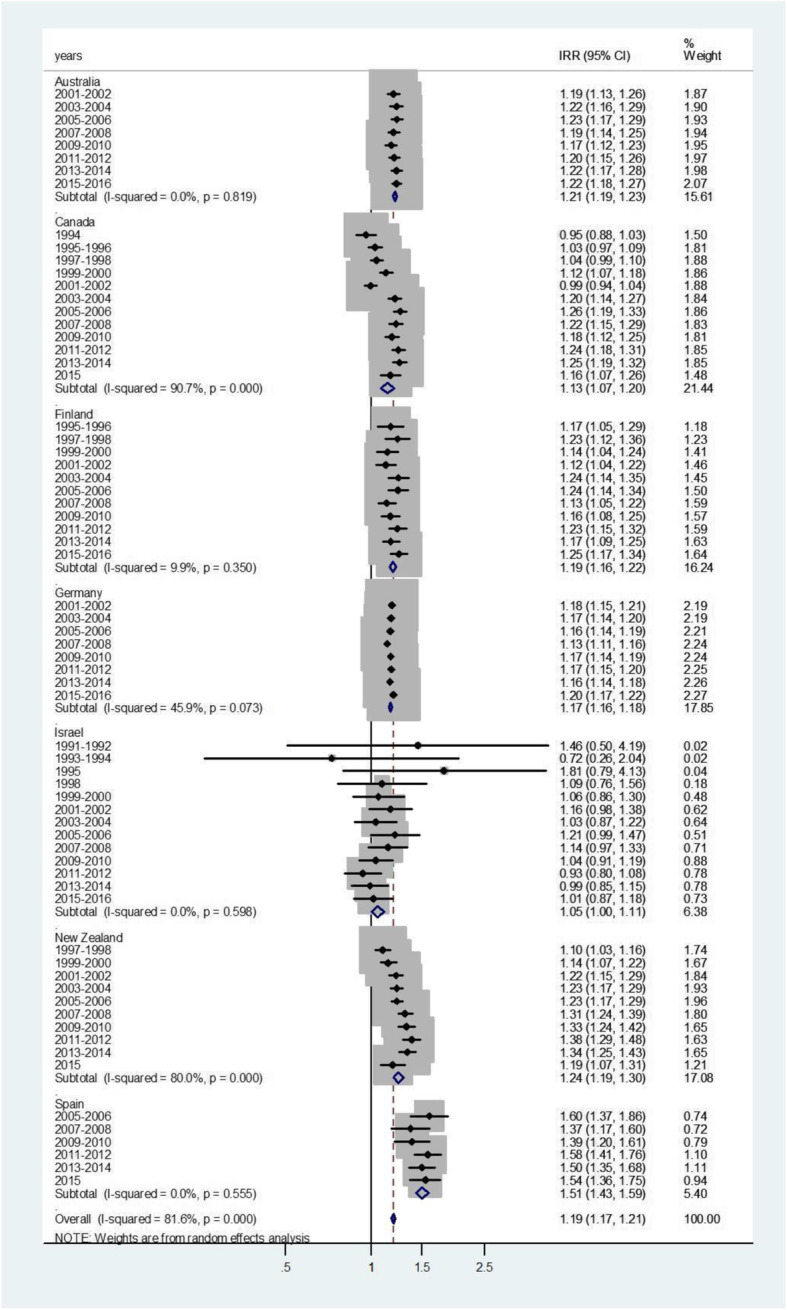
Forest plot of the male to female campylobacteriosis IRR at ages 45–64 or 40–59, for different years, in Australia, Canada, Finland, Germany, Israel, New Zealand, and Spain

**Fig. 7:**
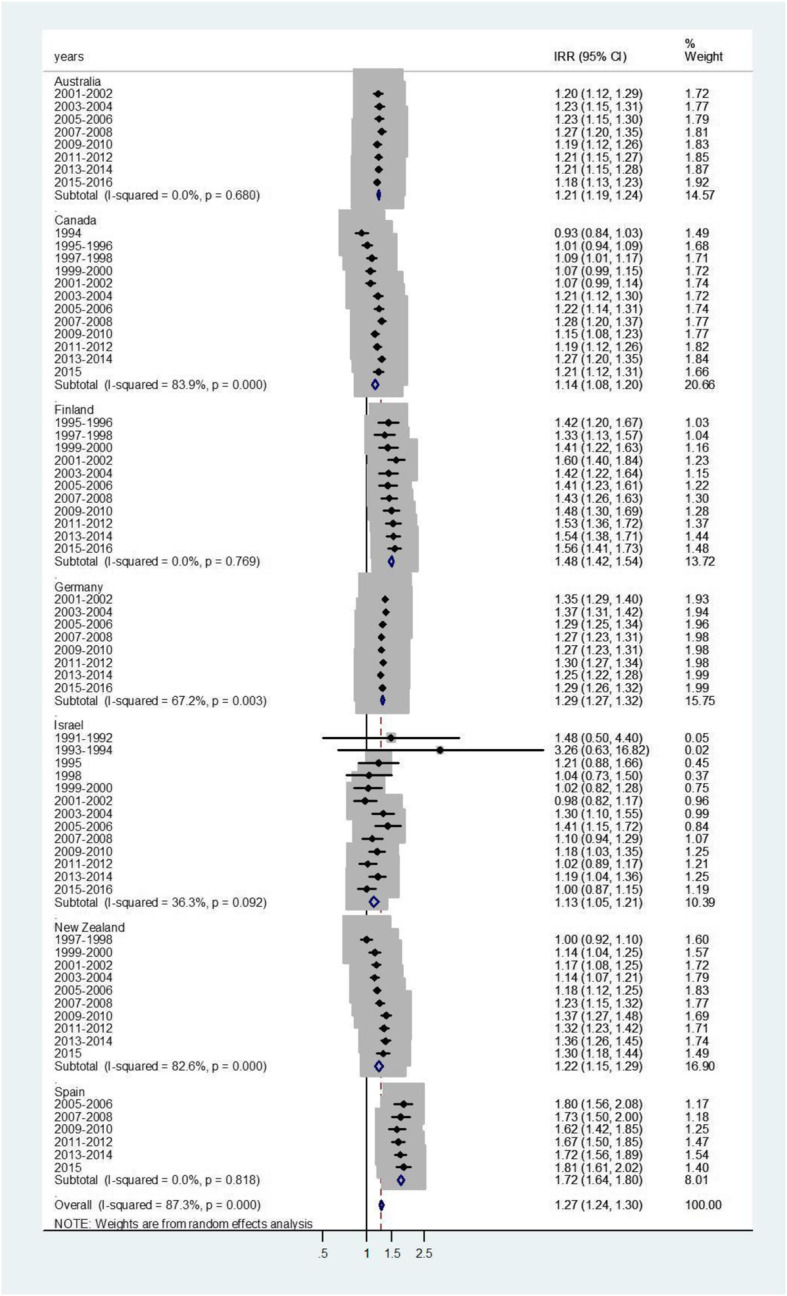
Forest plot of the male to female campylobacteriosis IRR at ages 65+ or 60+, for different years, in Australia, Canada, Finland, Germany, Israel, New Zealand, and Spain

The results of the meta-analyses are presented for the in Figs. 1, 2, 3, 4, 5, 6 and 7. For all age groups and countries, there was an excess in incidence rate in males. In some there there were quantitative differences between the countries, illustrated by the relatively high I^2^ values. This appeared to be influenced largely by the results from Israel, where the excess in incidence rates for males was highest for most age groups. We have no explanation for this.

The forest plot of the male to female campylobacteriosis IRRs for infants (< 1 year), for different years and countries is shown in Fig. 1. There was a 31% excess in the overall pooled incidence rate for males and the pooled IRRs varied from 1.21 in Germany to 1.52 in Israel with overall I^2^ = 67%.

The forest plot of the male to female campylobacteriosis IRRs at ages 1–4, for different years, is shown in Fig. 2. There was a 34% excess in the overall pooled incidence rate in males, and the pooled IRRs varied between 22% in Germany and 51% in Israel with overall I^2^ = 80.3%.

The forest plot of the male to female campylobacteriosis IRRs at ages 5–9, for different years, is shown in Fig. 3. In the age group 5–9, There was a 35% excess in the overall pooled incidence rate in males, with pooled IRRs varying between 21% in Germany and 47% in Israel with overall I^2^ = 66%.

The forest plot of the male to female campylobacteriosis IRRs at ages 10–14, for different years, is shown in Fig. 4. There was a 73% excess the overall pooled incidence rate in males, and the IRRs varied between 47% in Finland to more than double in males in Israel (overall I^2^ = 72.9%).

The forest plot of the male to female campylobacteriosis IRRs at ages 15–44 or 15–39), for different years, is shown in Fig. 5. Males had a 10% excess in incidence rates. The pooled IRRs varied from no excess in Germany to 19% in Spain (overall I^2^ = 94%).

The forest plot of the male to female campylobacteriosis IRRs at ages 45–64 or 40–59, for different years, is shown in Fig. 6. Males had a 19% excess in incidence rates. The pooled IRRs varied from a 5% excess in Israel to a 51% excess in Spain (overall I^2^ = 81%).

The forest plot of the male to female campylobacteriosis IRRs at ages 65+ or 60+, for different years, is shown in Fig. 7. Males had a 27% excess in incidence rates and ranged from 13% in Israel to 72% in Spain (overall I^2^ = 87.3%).

### Sensitivity analysis

To evaluate the effect of individual countries and years on the pooled IRR, we performed leave-one-out sensitivity analysis and recomputed the pooled IRRs. After omitting one country at a time, the pooled IRRs remained very similar (Table A2, Appendix A). Similar results were obtained after omission of several groups of years at a time (Table A3, Appendix A) Thus, no single country or particular groups of years substantially influenced the pooled IRRs. This confirms that the results of this study are stable and robust.

### Meta-regression analysis

Meta-regression results revealed that the age groups (*p* < 0.0001) contributed to almost all the source of heterogeneity, with very little contributed by countries or years. There was no significant difference in the pooled IRR between infancy to early/ late childhood, and senior adulthood (*p* > 0.05).

### Asymmetry analysis

The Egger’s test for asymmetry was not significant for infants (*p* = 0.427), middle adulthood (*p* = 0.234), and senior adulthood (*p* = 0.746). Evidence of asymmetry was observed for the early and late childhood, puberty and young adulthood with *p* < .0001 (Fig. B1, Appendix B), suggesting some evidence of possible bias. We do not have any explanation for this, and it could be simply a chance finding.

## Discussion

In the present meta-analytic study of national data from seven countries, over a period of 11–26 years, we found that the incidence rates for clinically manifested campylobacteriosis were 31, 34, 35 and 73% higher in males in infancy, young and late childhood and puberty, respectively. In young, older and senior age adults, they were 10, 19 and 27% higher in males. These results findings are remarkably consistent over countries and over a number of years. Our findings considerably extend those from population-based studies in multiple or single countries [10–12] and provide age-specific pooled estimates of the male to female IRR, while controlling for country and different time-periods. We have used the same meta-analytic methods to combine data from various countries and time periods for other diseases and demonstrated a male predominance in viral meningitis and shigellosis in young children [13, 14]. On the other hand, the incidence rates for pertussis were higher in females at all ages [29].

A major strength of the study is that it is based on national data with a relatively large numbers of cases. In addition, incidence rates based on populations which are reliable denominators. Selection bias has been minimized by using national data over different time periods, which should be representative of each country. The inclusion of seven countries, with data analysed over a number of years has permitted us to evaluate the consistency of the findings. We do not believe that excluding countries that have poor diagnostic facilities or reporting of infectious diseases should affect the sex differences in incidence rates, although other unknown factors might impact on the manifestations of the diseases in other countries. Since the clinical manifestations of campylobacteriosis vary widely, there could be significant under-reporting, but this should not differ between the sexes. Finally, differences in laboratory methods, either within or between countries, are unlikely to be related to the sex of the patients. Seasonal variation in the incidence of campylobacteriosis is well-documented with higher rates in the summer months [30]. However, there is no reason to suspect that differences in warmer and cooler countries would impact on the sex differences.

As in other studies where we have used this methodology to identify consistencies in sex differences in infectious diseases [13, 14, 29], however, in this study we cannot address the exact mechanisms underlying the sex differences in the incidence of clinical campylobacteriosis. There are a number of possibilities that have been postulated about the potential roles of cultural, behavioural, genetic, hormonal factors and microbiota [31–33]. Regarding possible cultural factors, in the countries in this study, there is no evidence that the sex of the child influences seeking for medical care for acute infections. Similarly, there is no evidence to suggest that in these countries, adult men are more likely than women to seek medical care for acute conditions of comparable severity although there is some evidence that women use health services more than men do [34]. Sex differences in exposure due to behavioural factors are unlikely to play a part in infants and very young children. It is likely that women spend more time caring for young children, this could increase their risk of infection. However, transmission of Campylobacter is mainly through food and water, and person-to person spread has not been found to be common [1, 3, 5].

In the older age groups, males may be more likely to be exposed as a result of consumption of inadequately cooked food eaten outside of the home [35]. The use of proton pump inhibitors is a factor that could influence the incidence of the disease in older people [36]. However, this should not impact on the sex differences in the disease incidence unless there are differences between men and women in the use of proton pump inhibitors.

As regards genetic factors, the humoral and cell-mediated immune responses appear to be stronger in females and since the X chromosomes contain genes associated with immune system, this could be an important factor in the immune response to Campylobacter infection [37]. Sex hormones could play an important role. Estradiol promotes innate immune signalling pathways and can enhance production of pro-inflammatory cytokines and chemokines in response to TLR (Toll-like receptor) ligand stimulation of dendritic cells and macrophages [38, 39]. This could partly explain the more effective immune response among females. In addition, testosterone can depress the innate and adaptive immune response [40], increasing the male susceptibility to clinical disease.

All or some of these factors could influence the mechanism of infection by Campylobacter. It has been shown that Campylobacter interferes with host innate immune signalling and the flagellins, FlaA and FlaB have been found to activate the innate immune receptor Toll-like receptor 5 (TLR5) [41]. Al-Banna et al. [6] have proposed that the immune response induced by Campylobacter induces a cascade of pro-inflammatory cytokines initiated by intestinal epithelial cells and innate cells, promoted by antigen-presenting cells and enhanced by T cells, but resolved by anti-inflammatory cytokines. Some or all of these factors could modify the immune response to infection and contribute to the sex differences in the incidence rates from campylobacteriosis.

In animal studies, Zeng et al. [8] recently demonstrated that innate antibodies against enteropathogenic *Escherichia coli* (EPEC) were present only in female mice after puberty and developed as a response to estrogen. They showed that these antibodies enabled Kupffer cells to capture circulating EPEC and were not dependent on previous exposure to the antigen. Thus, differences in sex hormone levels could play a significant biological role in the immune response to infection with Campylobacter and result in higher incidence rates of campylobacteriosis in males.

In the first year of life, sex hormone levels differ between males and females during the so-called “mini-puberty”. It is characterized by higher testosterone levels in boys at 1–3 months of age which decline at 6–9 months of age, whereas in girls, estradiol levels remain elevated longer [42]. Thus, sex hormones can affect immune cells in the first year of life and perhaps even later. It is of interest to note that the largest excess in incidence rates for males was in the age 10–14, where both hormonal and behavioural factors could be operating.

Microbiota can affect immunity by direct interaction with immune cells, by epigenetic modification and via the production of signaling biomolecules. Recent studies indicate sex-specific differences in immune responses based on the gut microbiota associated microorganisms that significantly influence the function of innate and adaptive immunity [33, 43].

As mentioned earlier, a serious complication of campylobacteriosis is the Guillain–Barré syndrome. It is interesting to note that there are reports that the Guillain Barré syndrome is also more common in males [44]. It is not clear whether the excess incidence of Guillain–Barré syndrome in males is confined solely to those cases occurring as result of campylobacteriosis.

## Conclusions

In conclusion, the remarkably consistent excess incidence of campylobacteriosis in males, particularly in infants and very young children, suggests and that inherent sex-specific factors and not just exposure differences influence the incidence of clinical disease. These findings should stimulate research on sex as a biological variable in the pathogenesis of campylobacteriosis.

## Supplementary information

**Additional file 1.** Appendix A

**Additional file 2.** Appendix B

## Data Availability

All data are available from the original sources or from the authors. For all countries, except Israel, public access to the databases is open. We received administrative permission from the official representative of Israeli Ministry of Health to use the data for publication. Links and references: •National Notifiable Diseases Surveillance System (NNDSS), Department of Health: http://www9.health.gov.au/cda/source/cda-index.cfm. Accessed on April 1, 2018. •Public Health Agency of Canada: https://www.canada.ca/en/public-health.html. Accessed on June 1, 2018. •National institute for health and welfare (THL): https://www.thl.fi/ttr/gen/rpt/tilastot.html. Accessed on May 1, 2018. •German Federal Health Monitoring System: http://www.gbe-bund.de/gbe10/pkg_isgbe5.prc_isgbe?p_uid=gast&p_aid=0&p_sprache=D. Accessed on February 1, 2018. •Environmental Science and Research (ESR) for the Ministry of Health: https://surv.esr.cri.nz/surveillance/annual_surveillance.php. Accessed on March 30, 2018. •Instituto de Salud Carlos III (Informes anuales RENAVE):https://www.isciii.es/QueHacemos/Servicios/VigilanciaSaludPublicaRENAVE/EnfermedadesTransmisibles/Paginas/Informes-anuales-RENAVE.aspx. Some of the data can be obtained by searching the web. Keyword searched: RESULTADOS DE LA VIGILANCIA EPIDEMIOLÓGICA DE LAS ENFERMEDADES TRANSMISIBLES. INFORME ANNUAL. Accessed on March 1, 2018. •ABS.Stat (Australian Bureau of Statistics): http://stat.data.abs.gov.au/Index.aspx? DatasetCode = ABS_ERP_ASGS2016.Accessed on May 15, 2018. •Statistics, Canada, CANSIM database: https://www150.statcan.gc.ca/t1/tbl1/en/cv.action?pid=1710010201. Accessed on June 1, 2018. •Statistics Finland’s PX-Web databases: https://pxnet2.stat.fi/PXWeb/pxweb/en/StatFin/. Accessed on April 15, 2018. •German Federal Health Monitoring System: http://www.gbe-bund.de/gbe10/abrechnung.prc_abr_test_logon?p_uid=gast&p_aid=46300054&p_knoten=VR&p_sprache=E&p_suchstring=population. Accessed on February 1, 2018. •Central Bureau of Statistics: http://www.cbs.gov.il/reader/shnatonhnew_site.htm?sss=%E4%EE%F9%EA&shnaton_scan=45. Accessed on March 1, 2018. •Stats NZ, Infoshare: http://archive.stats.govt.nz/infoshare/SelectVariables.aspx?pxID=b854d8a2-3fdf-402c-af69-604112e80baa. Accessed on May 15, 2018. •Demographic Statistics Database (United Nations Statistics: Division): http://data.un.org/Data.aspx?d=POP&f=tableCode%3A22. Accessed on April 1, 2018.
