# Supplementary material for: Sex differences in campylobacteriosis incidence rates at different ages - a seven country, multi-year, meta-analysis. A potential mechanism for the infection
Source: BMC Infect Dis. 2020 Aug 25;20:625. doi: 10.1186/s12879-020-05351-6 (PMC7445732; doi:10.1186/s12879-020-05351-6)
Supplement: Supplementary file 1 — Additional file 1. Appendix A [file 12879_2020_5351_MOESM1_ESM.docx]

Appendix A

Table legends

Table A1: Details of the countries included in the meta-analysis, by sex and age group – total cases, population size, incidence rates (IR) per 100 000 and IRR( male : female Incidence Rate Ratio).

Table A2: Sensitivity analysis of IRR’s for each age group, by removing one country at a time. (RR = rate ratio; CI = confidence interval)

Table A3: Sensitivity analysis of IRR’s for each age group, by removing group of years at a time. (RR = rate ratio; CI = confidence interval)

**Table A1:** Details of the countries included in the meta-analysis, by sex and age group – total cases, population size, incidence rates (IR) per 100 000 and incidence RR( male : female Incidence Rate Ratio).

|  |  | |  | | | | | | | | **Males** | | | | **Females** | | | | | **IRR** | | |
| --- | --- | --- | --- | --- | --- | --- | --- | --- | --- | --- | --- | --- | --- | --- | --- | --- | --- | --- | --- | --- | --- | --- |
| **Age group** | | **Country** | | | **Years** | | | | | | **n/N** | | **IR** | | **n/N** | | | | **IR** | | |  |
| **<1** | | Canada | | | | | | 1994-2015 | | | 2045/4066314 | | 50.3 | | 1539/3861381 | | | 39.9 | | | 1.3 | |
|  | | Germany | | | | | | 2001-2016 | | | 4786/5740478 | | 83.4 | | 3768/5448550 | | | 69.2 | | | 1.2 | |
|  | | Israel | | | | | | 1991-2016 | | | 6669/1770100 | | 376.8 | | 4151/1680000 | | | 247.1 | | | 1.5 | |
|  | | New Zealand | | | | | | 1997-2015 | | | 2128/576900 | | 368.9 | | 1576/548520 | | | 287.3 | | | 1.3 | |
|  | | Spain | | | | | | 2005-2015 | | | 7054/2679186 | | 263.3 | | 5355/2514548 | | | 213 | | | 1.2 | |
|  |  | |  | | | | | | | |  | |  | |  | | |  | | |  | |
| **1-4** | | Canada | | | | | | | 1994-2015 | | 11480/16718349 | | 68.7 | | 8252/15900004 | | | 51.9 | | | 1.3 | |
|  | | Germany | | | | | | | 2001-2016 | | 30709/23509315 | | 130.6 | | 23847/22311030 | | | 106.9 | | | 1.2 | |
|  | | Israel | | | | | | | 1991-2016 | | 15607/6843500 | | 228.1 | | 9811/6500900 | | | 150.9 | | | 1.5 | |
|  | | New Zealand | | | | | | | 1997-2015 | | 10525/2308880 | | 455.9 | | 7520/2191980 | | | 343.1 | | | 1.3 | |
|  | | Spain | | | | | | | 2005-2015 | | 16687/10880587 | | 153.4 | | 12341/10233932 | | | 120.6 | | | 1.3 | |
|  |  | |  | | | | | | | |  | |  | |  | | |  | | |  | |
| **5-9** | | Australia | | | | | | 2001-2016 | | | 9146/11398585 | | 80.2 | | 6402/10814642 | | | 59.2 | | | 1.4 | |
|  | | Canada | | | | | | 1994-2015 | | | 7280/21678340 | | 33.6 | | 5004/20622712 | | | 24.3 | | | 1.4 | |
|  | | Finland | | | | | | 1995-2016 | | | 789/3440956 | | 23 | | 564/3297629 | | | 17.1 | | | 1.3 | |
|  | | Germany | | | | | | 2001-2016 | | | 22704/30760941 | | 73.8 | | 17873/29187252 | | | 61.2 | | | 1.2 | |
|  | | Israel | | | | | | 1991-2016 | | | 5138/7977400 | | 64.4 | | 3304/7580100 | | | 43.6 | | | 1.5 | |
|  | | New Zealand | | | | | | 1997-2015 | | | 5161/2899540 | | 178 | | 3540/2752910 | | | 128.6 | | | 1.4 | |
|  | | Spain | | | | | | 2005-2015 | | | 5512/13017097 | | 42.3 | | 3919/12287011 | | | 31.9 | | | 1.3 | |
|  |  | | | | | | | | |  |  | |  | |  | | |  | | |  | |
| **10-14** | | Australia | | | | | | 2001-2016 | | | 7816/11377822 | | 68.7 | | 4080/10797396 | | | 37.8 | | | 1.8 | |
|  | | Canada | | | | | | 1994-2015 | | | 5663/22713799 | | 25 | | 3124/21572803 | | | 14.5 | | | 1.7 | |
|  | | Finland | | | | | | 1995-2016 | | | 976/3522497 | | 27.7 | | 633/3375446 | | | 18.8 | | | 1.5 | |
|  | | Germany | | | | | | 2001-2016 | | | 24472/33455166 | | 73.1 | | 15176/31724889 | | | 47.9 | | | 1.5 | |
|  | | Israel | | | | | | 1991-2016 | | | 3822/7398300 | | 51.6 | | 1720/7029400 | | | 24.5 | | | 2.1 | |
|  | | New Zealand | | | | | | 1997-2015 | | | 4906/2919850 | | 168 | | 2579/2776650 | | | 92.9 | | | 1.8 | |
|  | | Spain | | | | | | 2005-2015 | | | 2382/12301238 | | 19.4 | | 1436/11627137 | | | 12.4 | | | 1.6 | |
|  |  | |  | | | | | | | |  | |  | |  | | |  | | |  | |
| **15-44**  **15** | | Australia | | | | | | | 2001-2016 | | 61143/73591102 | | 83.1 | | 54717/72741755 | | | 75.2 | | | 1.1 | |
| **(15-39)** | | Canada | | | | | | | 1994-2015 | | 52598/126619246 | | 41.5 | | 44735/123505034 | | | 36.2 | | | 1.1 | |
|  | | Finland | | | | | | | 1995-2016 | | 19972/18898064 | | 105.7 | | 18885/18050351 | | | 104.6 | | | 1.0 | |
|  | | Germany | | | | | | | 2001-2016 | | 245134/257895408 | | 95.1 | | 234556/247590330 | | | 94.7 | | | 1.0 | |
|  | | Israel | | | | | | | 1991-2016 | | 9426/35538900 | | 26.5 | | 8129/35142900 | | | 23.1 | | | 1.1 | |
|  | | New Zealand | | | | | | | 1997-2015 | | 37402/13546700 | | 276.1 | | 33501/13976900 | | | 239.7 | | | 1.2 | |
|  | | Spain | | | | | | | 2005-2015 | | 4881/110542308 | | 4.4 | | 3947/105413400 | | | 3.7 | | | 1.2 | |
|  |  | |  | | | | | | | |  | |  | |  | | |  | | |  | |
| **45-64** | | Australia | | | | | 2001-2016 | | | | 31795/41988401 | | 75.7 | | 26708/42573071 | | | 62.7 | | | 1.2 | |
| **(40-59)** | | Canada | | | | | 1994-2015 | | | | 31090/100585696 | | 30.9 | | 27041/99821361 | | | 27.1 | | | 1.1 | |
|  | | Finland | | | | | 1995-2016 | | | | 15233/16513241 | | 92.2 | | 12656/16307550 | | | 77.6 | | | 1.2 | |
|  | | Germany | | | | | 2001-2016 | | | | 131198/181698132 | | 72.2 | | 112386/181849520 | | | 61.8 | | | 1.2 | |
|  | | Israel | | | | | 1991-2016 | | | | 2708/14322400 | | 19 | | 2774/15453800 | | | 18 | | | 1.1 | |
|  | | New Zealand | | | | | 1997-2015 | | | | 23452/10201030 | | 230 | | 19708/10685350 | | | 184.4 | | | 1.2 | |
|  | | Spain | | | | | 2005-2015 | | | | 3394/63103755 | | 5.4 | | 2296/64340310 | | | 3.6 | | | 1.5 | |
|  |  | | |  | | | | | | | |  |  | | |  | |  | | |  | |
| **65/+60+** | | Australia | | | | 2001-2016 | | | | | | 20158/21417772 | 94 | | | 19725/25538457 | | 77.2 | | | 1.2 | |
|  | | Canada | | | | 1994-2015 | | | | | | 18731/58764646 | 31.9 | | | 19547/70995360 | | 27.5 | | | 1.2 | |
|  | | Finland | | | | 1995-2016 | | | | | | 5263/11159619 | 47.2 | | | 4752/15066114 | | 31.5 | | | 1.5 | |
|  | | Germany | | | | 2001-2016 | | | | | | 64640/108019284 | 59.8 | | | 68754/149862231 | | 45.9 | | | 1.3 | |
|  | | Israel | | | | 1991-2016 | | | | | | 2734/7087900 | 38.6 | | | 3194/9298400 | | 34.3 | | | 1.1 | |
|  | | New Zealand | | | | 1997-2015 | | | | | | 14760/6302700 | 234.2 | | | 14224/7386000 | | 192.6 | | | 1.2 | |
|  | | Spain | | | | 2005-2015 | | | | | | 3788/37127234 | 10.2 | | | 2948/49879431 | | 5.9 | | | 1.7 | |
|  | | | |  | | | | | | | |  | |  | | |  | | | | |  |

N- Cumulative total of the population for given years.

Table A2: Sensitivity analysis of IRR’s for each age group, by removing one country at a time

| **Age group**  **Country Removed** | **Infants**  **RR (CI)** | **Early Childhood**  **RR (CI)** | **Late childhood**  **RR (CI)** | **Puberty**  **RR (CI)** | **Young adulthood**  **RR (CI)** | **Middle adulthood**  **RR (CI)** | **Senior adulthood**  **RR (CI)** |
| --- | --- | --- | --- | --- | --- | --- | --- |
| **Australia** | - | - | 1.35  (1.25-1.45) | 1.69  (1.53-1.87) | 1.1  (1.03-1.18) | 1.21  (1.15-1.26) | 1.32  (1.21-1.44) |
| **Canada** | 1.31  (1.17-1.46) | 1.33  (1.21-1.46) | 1.34  (1.25-1.44) | 1.71  (1.54-1.9) | 1.1  (1.03-1.16) | 1.22  (1.17-1.27) | 1.33  (1.24-1.43) |
| **Finland** | - | - | 1.35  (1.26-1.44) | 1.75  (1.59-1.92) | 1.12  (1.05-1.19) | 1.21  (1.16-1.26) | 1.27  (1.19-1.37) |
| **Germany** | 1.32  (1.18-1.48) | 1.35  (1.25-1.46) | 1.38  (1.34-1.42) | 1.75  (1.62-1.89) | 1.12  (1.08-1.16) | 1.21  (1.15-1.28) | 1.3  (1.19-1.43) |
| **Israel** | 1.24  (1.21-1.27) | 1.28  (1.23-1.34) | 1.33  (1.25-1.41) | 1.65  (1.53-1.79) | 1.1  (1.03-1.17) | 1.23  (1.18-1.27) | 1.34  (1.24-1.44) |
| **New**  **Zealand** | 1.3  (1.16-1.46) | 1.33  (1.21-1.45) | 1.34  (1.25-1.41) | 1.69  (1.53-1.79) | 1.1  (1.03-1.17) | 1.2  (1.15-1.27) | 1.32  (1.22-1.44) |
| **Spain** | 1.31  1.16-1.49)) | 1.34  (1.22-1.48) | 1.35  (1.26-1.44) | 1.74  (1.57-1.87) | 1.09  1.03-1.16)) | 1.17  (1.14-1.25) | 1.25  (1.18-1.43) |

(IRR = incidence rate ratio; CI = confidence interval)

Table A3: Sensitivity analysis of IRR’s for each age group, by removing group of years at a time

| **Age group**  **Years removed** | **Infants** | **Early childhood** | **Late childhood** | **Puberty** | **Young adulthood** | **Middle adulthood** | **Senior adulthood** |
| --- | --- | --- | --- | --- | --- | --- | --- |
| 1991-1992 | 1.31 (1.28-1.34) | 1.3 (1.29-1.32) | 1.3 (1.28-1.33) | 1.65 (1.62-1.68) | 1.05 (1.04-1.06) | 1.16 (1.14-1.18) | 1.23 (1.2-1.27) |
| 1993-1994 | 1.31 (1.28-1.34) | 1.31 (1.29-1.32) | 1.3 (1.28-1.32) | 1.65 (1.62-1.68) | 1.05 (1.04-1.06) | 1.17 (1.15-1.19) | 1.25 (1.22-1.28) |
| 1995-1996 | 1.31 (1.28-1.34) | 1.31 (1.29-1.33) | 1.31 (1.28-1.33) | 1.65 (1.62-1.68) | 1.05 (1.03-1.06) | 1.17 (1.15-1.19) | 1.25 (1.22-1.28) |
| 1997-1998 | 1.31 (1.28-1.35) | 1.3 (1.29-1.32) | 1.3 (1.28-1.33) | 1.65 (1.62-1.68) | 1.05 (1.03-1.06) | 1.17 (1.15-1.19) | 1.25 (1.22-1.28) |
| 1999-2000 | 1.3 (1.28-1.33) | 1.3 (1.29-1.32) | 1.3 (1.28-1.32) | 1.65 (1.63-1.68) | 1.05 (1.04-1.06) | 1.16 (1.14-1.18) | 1.24 (1.21-1.28) |
| 2001-2002 | 1.31 (1.27-1.35) | 1.31 (1.29-1.33) | 1.31 (1.29-1.33) | 1.66 (1.63-1.69) | 1.05 (1.03-1.06) | 1.16 (1.14-1.18) | 1.23 (1.19-1.27) |
| 2003-2004 | 1.31 (1.27-1.34) | 1.3 (1.28-1.32) | 1.31 (1.28-1.33) | 1.65 (1.62-1.67) | 1.05 (1.03-1.06) | 1.15 (1.13-1.18) | 1.23 (1.19-1.26) |
| 2005-2006 | 1.31 (1.28-1.35) | 1.3 (1.28-1.32) | 1.3 (1.28-1.33) | 1.64 (1.62-1.67) | 1.05 (1.04-1.06) | 1.15 (1.13-1.18) | 1.23 (1.19-1.26) |
| 2007-2008 | 1.31 (1.27-1.35) | 1.31 (1.29-1.33) | 1.31 (1.29-1.33) | 1.66 (1.63-1.69) | 1.05 (1.04-1.06) | 1.16 (1.13-1.18) | 1.23 (1.19-1.26) |
| 2009-2010 | 1.31 (1.28-1.35) | 1.31 (1.29-1.33) | 1.31 (1.28-1.33) | 1.65 (1.62-1.68) | 1.05 (1.04-1.06) | 1.16 (1.13-1.18) | 1.23 (1.19-1.27) |
| 2011-2012 | 1.31 (1.27-1.34) | 1.31 (1.29-1.33) | 1.31 (1.28-1.33) | 1.65 (1.62-1.68) | 1.05 (1.03-1.06) | 1.15 (1.13-1.18) | 1.23 (1.19-1.26) |
| 2013-2014 | 1.3 (1.27-1.33) | 1.3 (1.28-1.32) | 1.31 (1.28-1.33) | 1.66 (1.63-1.68) | 1.05 (1.03-1.06) | 1.15 (1.13-1.18) | 1.23 (1.19-1.26) |
| 2015-2016 | 1.31 (1.28-1.35) | 1.31 (1.29-1.33) | 1.3 (1.28-1.32) | 1.65 (1.62-1.68) | 1.05 (1.04-1.06) | 1.15 (1.13-1.18) | 1.23 (1.19-1.26) |

(IRR = incidence rate ratio; CI = confidence interval)
