# Supplementary figures and images for: Sex differences in campylobacteriosis incidence rates at different ages - a seven country, multi-year, meta-analysis. A potential mechanism for the infection
Source: BMC Infect Dis. 2020 Aug 25;20:625. doi: 10.1186/s12879-020-05351-6 (PMC7445732; doi:10.1186/s12879-020-05351-6)

Additional file, Appendix B

Figure B1, Asymmetry analysis

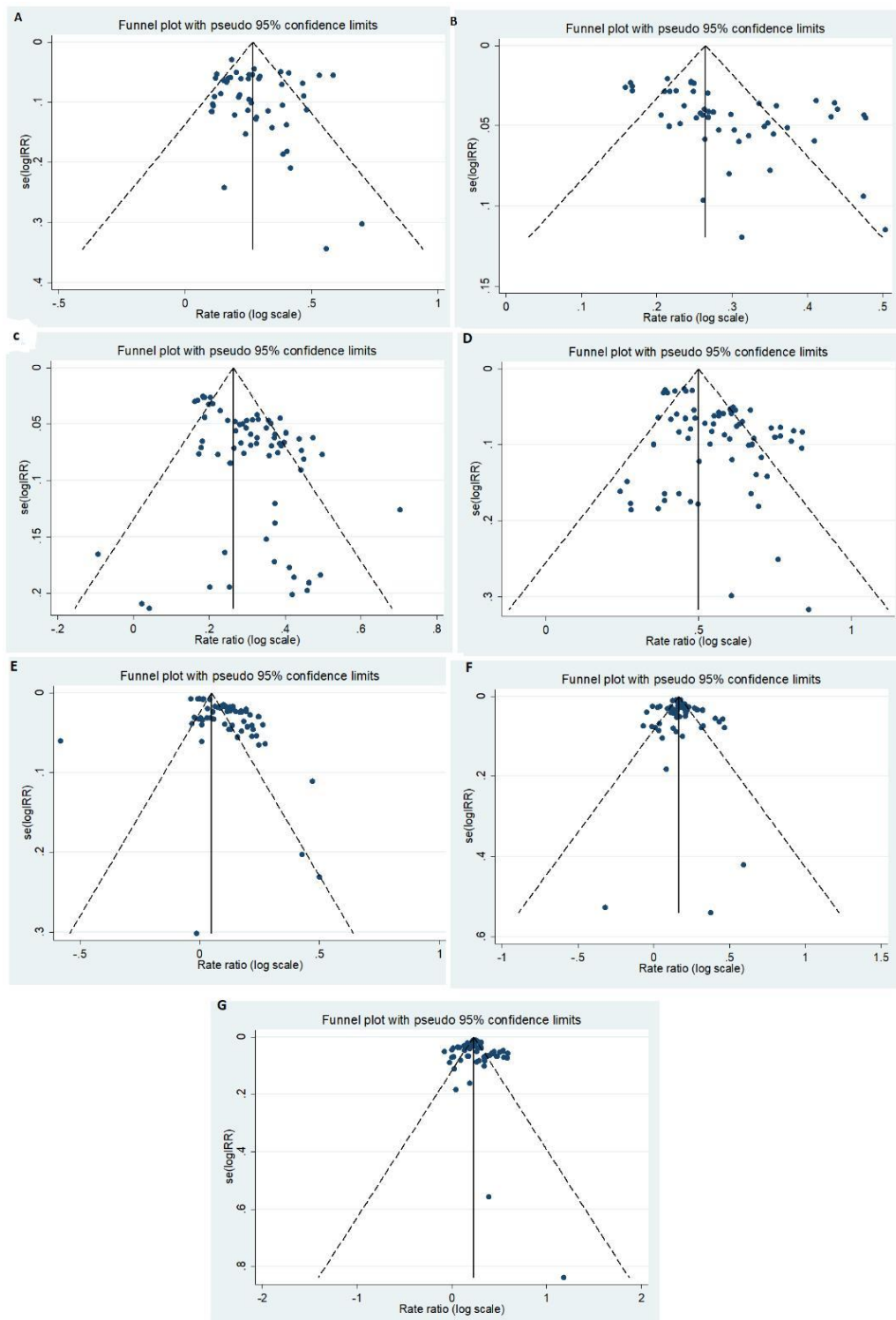

Supplement: Supplementary file 2 — Additional file 2. Appendix B [file 12879_2020_5351_MOESM2_ESM.pdf]
